# Supplementary material for: Harmonizing multisite neonatal diffusion-weighted brain MRI data for developmental neuroscience
Source: Dev Cogn Neurosci. 2024 Dec 8;71:101488. doi: 10.1016/j.dcn.2024.101488 (PMC11683243; doi:10.1016/j.dcn.2024.101488)
Supplement: Supplementary file 1 — Supplementary material [file mmc1.docx]

**Supplementary Figures**





**Supplementary figure 1.** Density plots of FA (raw values: A; after harmonization: B), MD (C, D), AD (E, F) and RD (G, H) before and after harmonization.


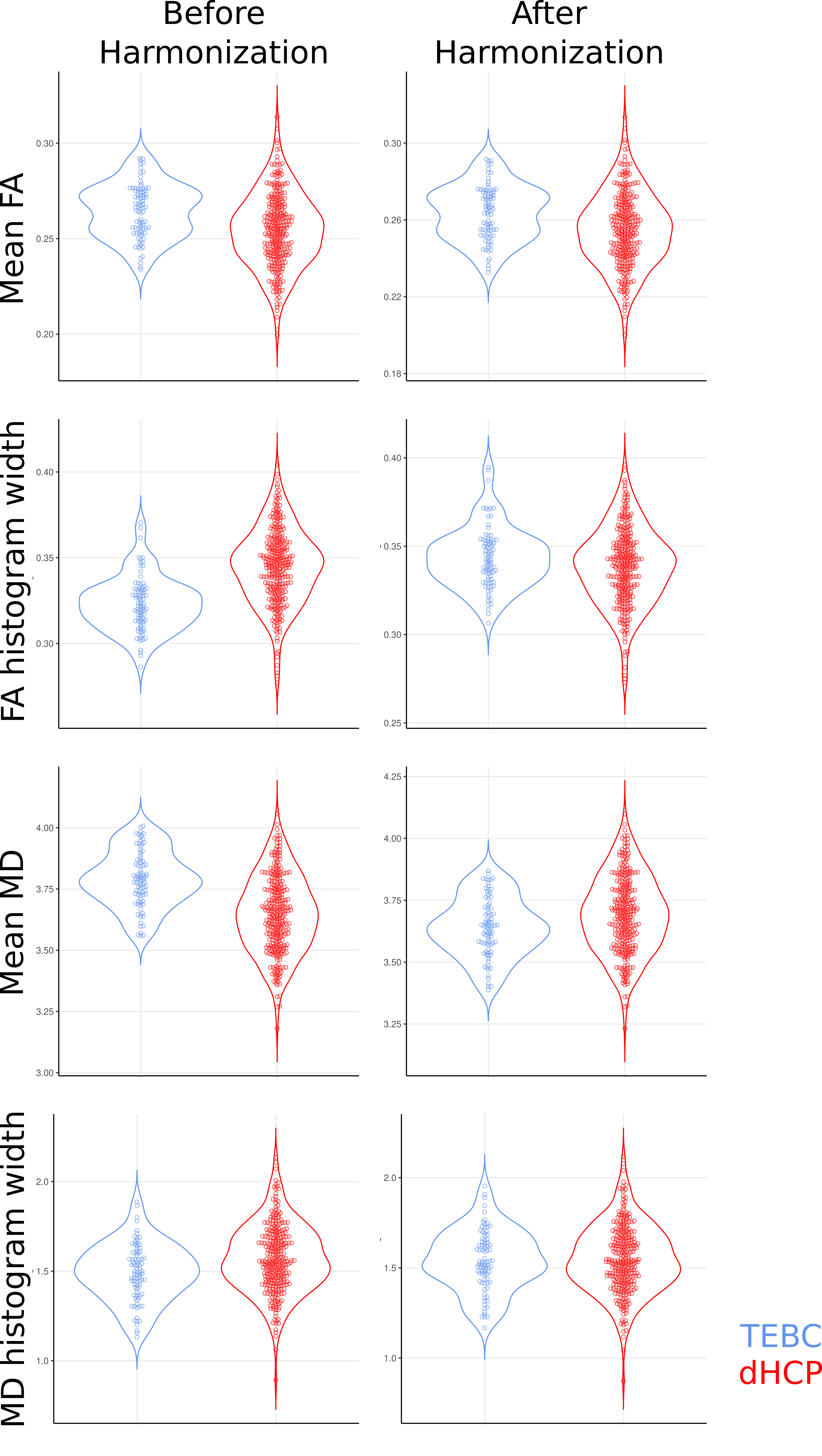


**Supplementary Figure 2.** Violin plots of mean values and histogram widths for FA and MD before and after harmonization in Theirword Edinburgh Birth Cohort (TEBC) and developing human connectome project (dHCP)


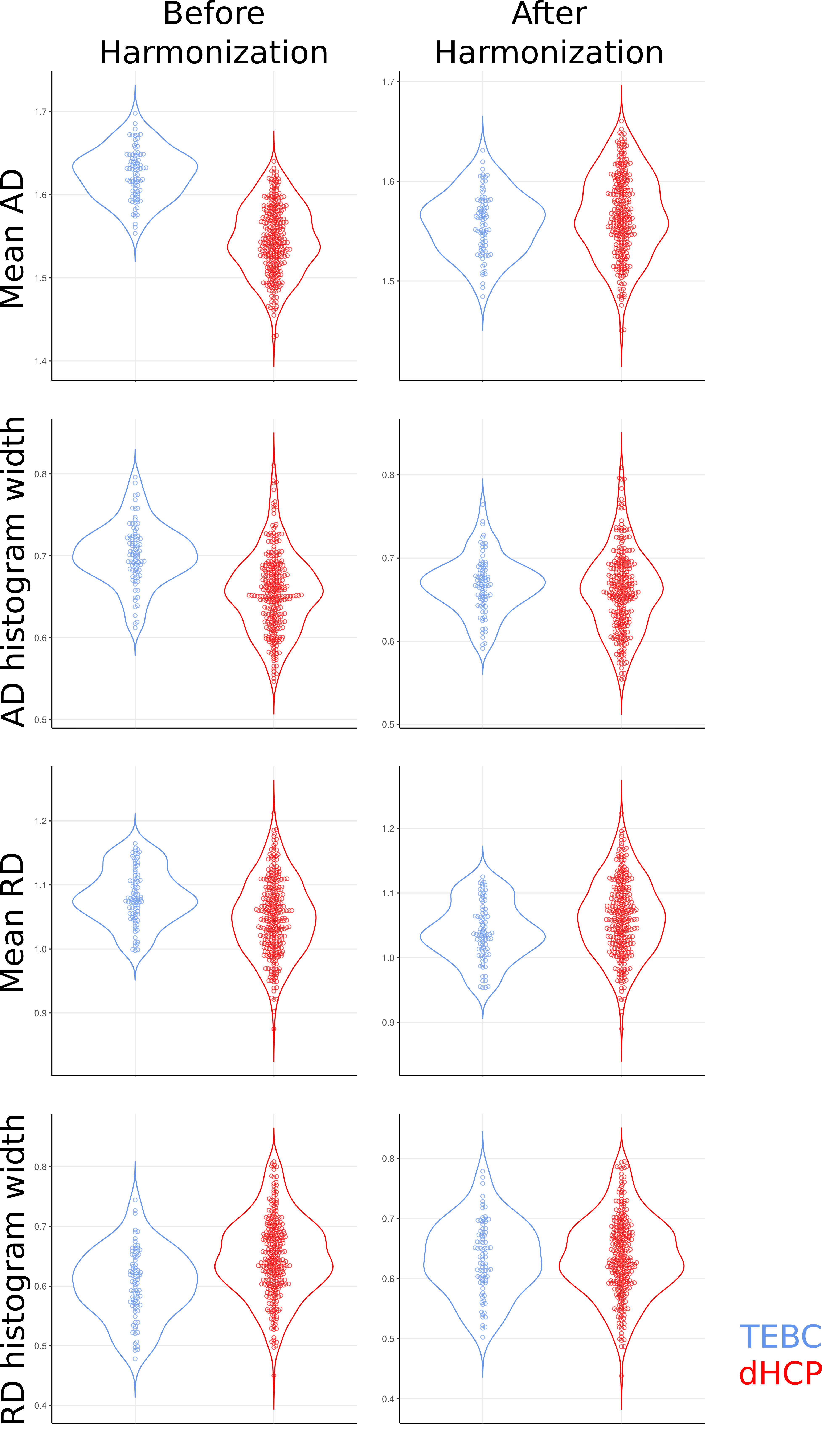


**Supplementary Figure 3.** Violin plots of mean values and histogram widths for AD and RD before and after harmonization in Theirword Edinburgh Birth Cohort (TEBC) and developing human connectome project (dHCP)


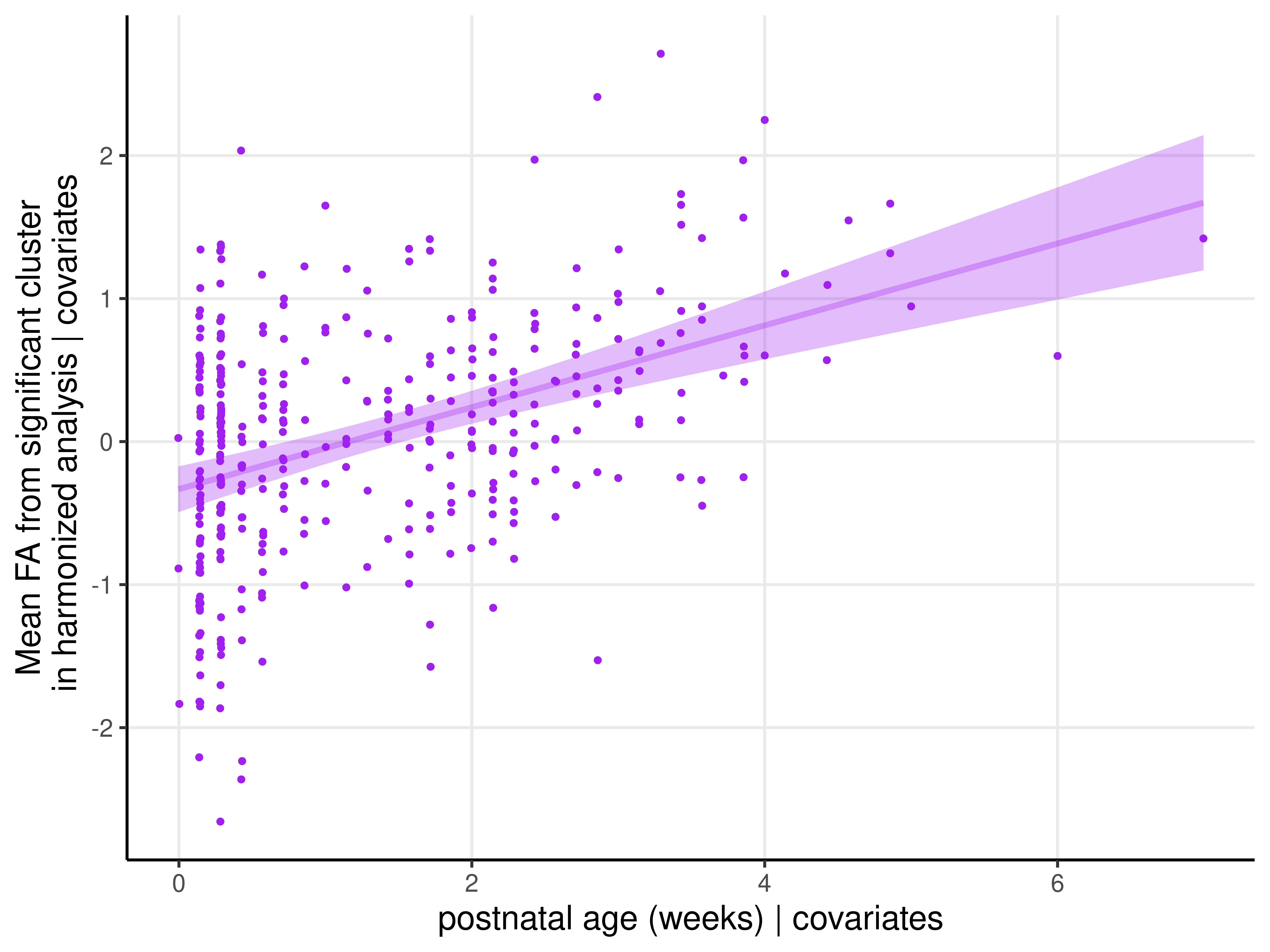


**Supplementary Figure 4.** Post-hoc analysis of relationship between FA values in the cluster within the left retrolenticular part of the internal capsule and postnatal age in weeks (β[SE]=0.358[0.053], p<0.001), adjusting for GA at scan and sex.


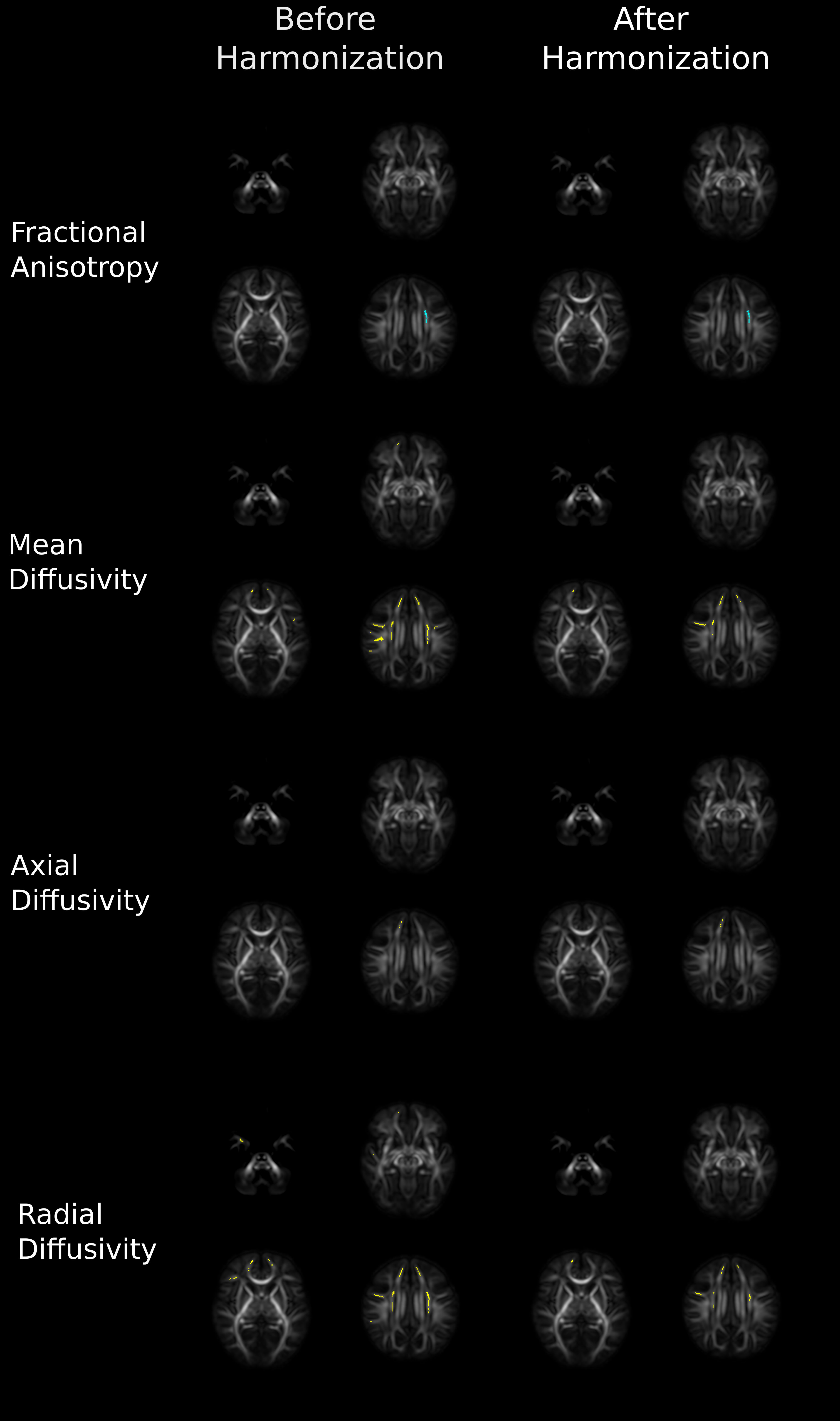


Supplementary Figure 5. Voxel-wise correlations with gestational age at birth in the Theirworld Edinburgh Birth Cohort before and after harmonization. Results represent voxels significantly positively (blue) and negatively (yellow) associated with gestational age at scan adjusting for sex and gestational age at scan.


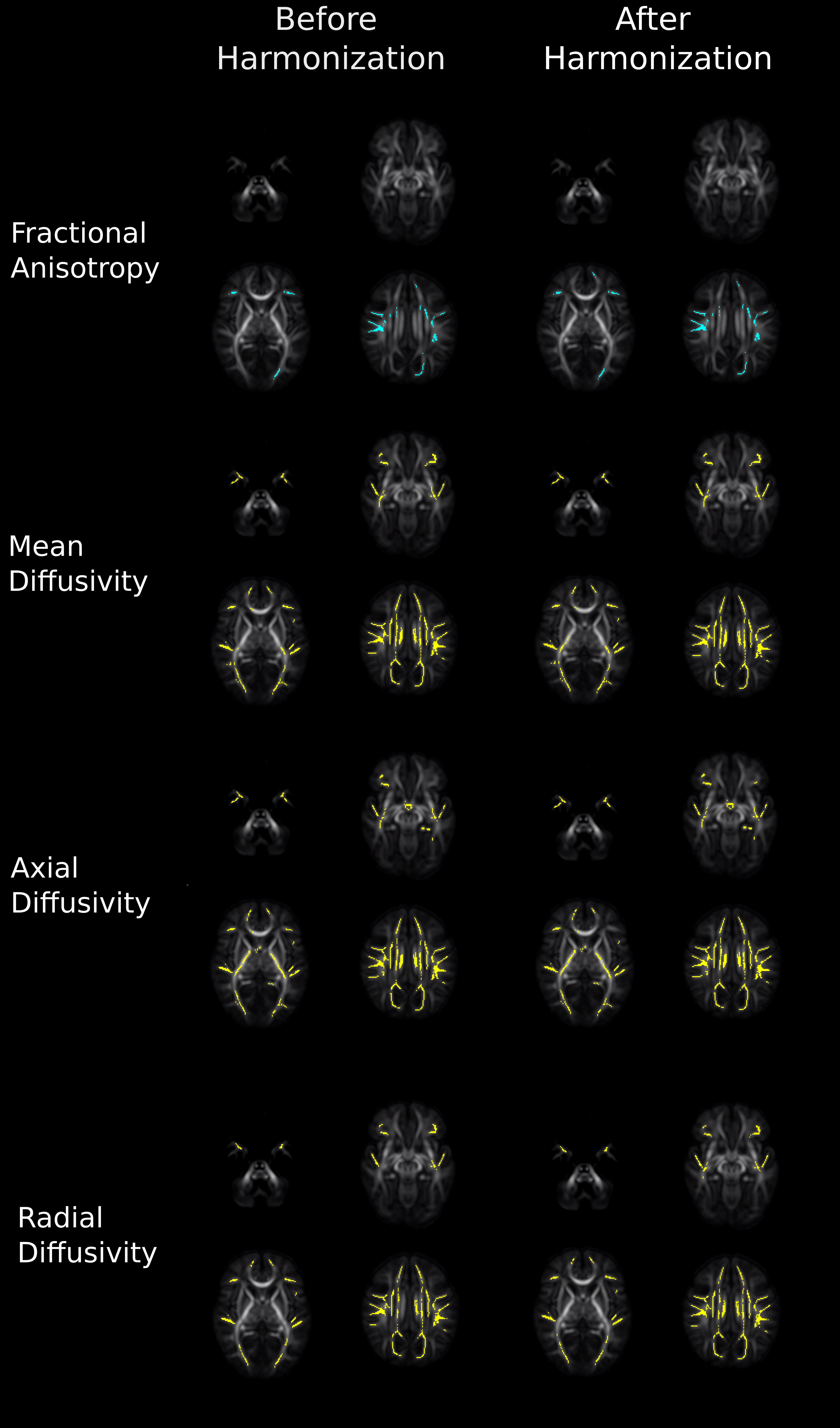


Supplementary Figure 6. Voxel-wise correlations with gestational age at birth in the Developing Human Connectome Project before and after harmonization. Results represent voxels significantly positively (blue) and negatively (yellow) associated with gestational age at scan adjusting for sex and gestational age at scan.


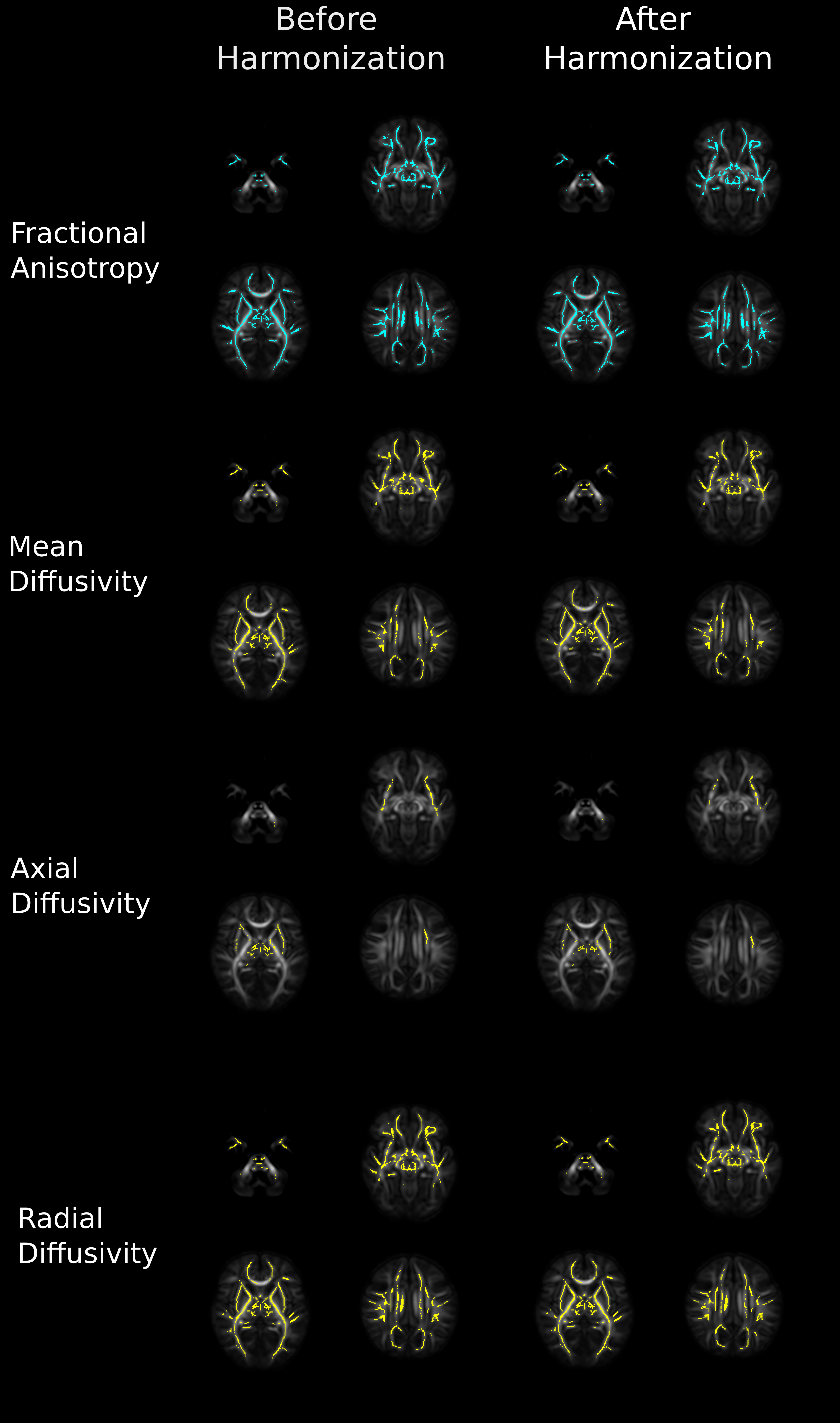


Supplementary Figure 7. Voxel-wise correlations with gestational age at scan in the Theirworld Edinburgh Birth Cohort before and after harmonization. Results represent voxels significantly positively (blue) and negatively (yellow) associated with gestational age at scan adjusting for sex and gestational age at birth.


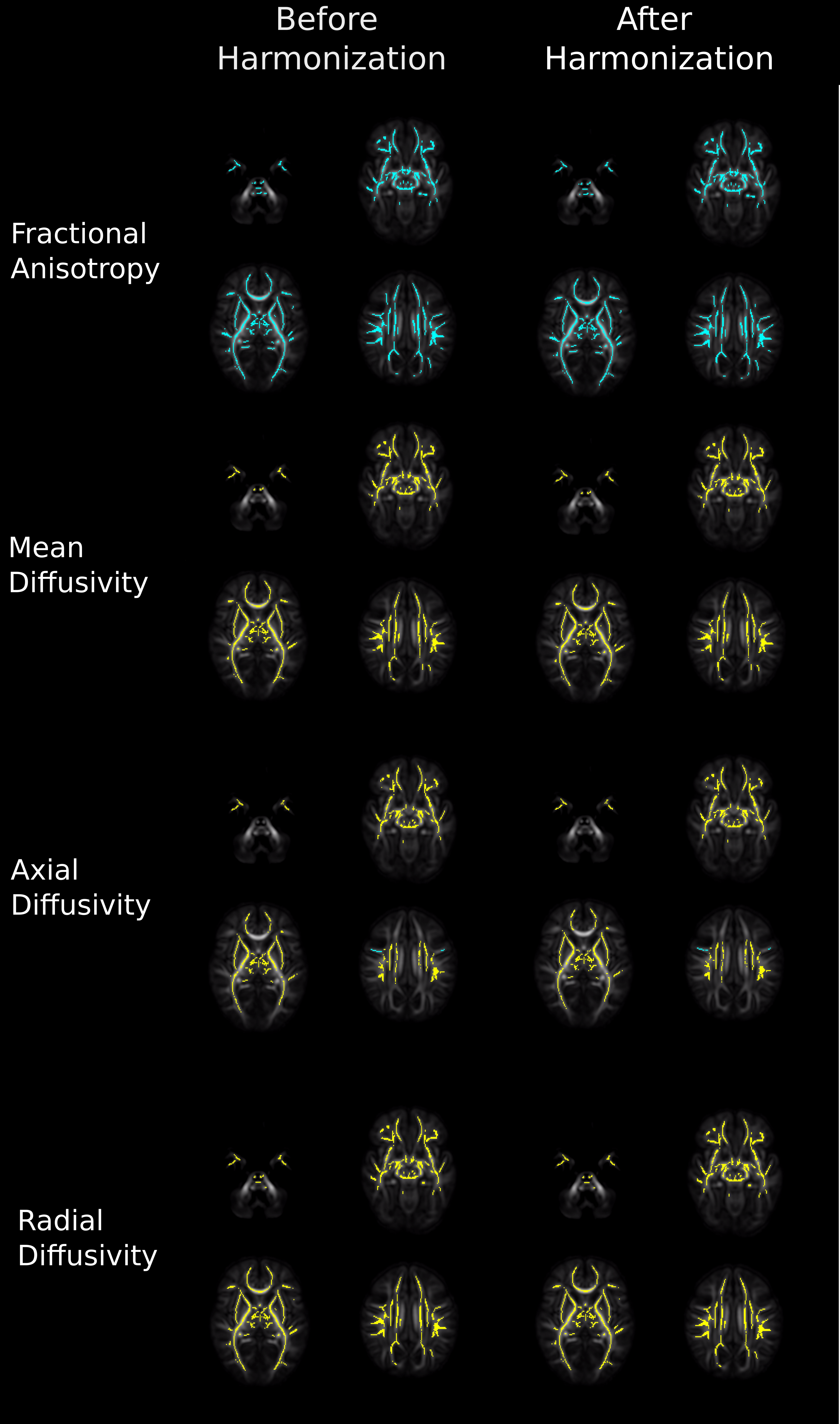


Supplementary Figure 8. Voxel-wise correlations with gestational age at scan in the Developing Human Connectome Project before and after harmonization. Results represent voxels significantly positively (blue) and negatively (yellow) associated with gestational age at scan adjusting for sex and gestational age at birth.
